# Supplementary material for: Continual familiarity decoding from recurrent connections in spiking networks
Source: PLoS Comput Biol. 2025 Aug 1;21(8):e1013304. doi: 10.1371/journal.pcbi.1013304 (PMC12334059; doi:10.1371/journal.pcbi.1013304)
Supplement: S1 Table — Values of model and connectivity parameters fixed in all experiments. (DOCX) [file pcbi.1013304.s001.docx]

## S1 Table. Fixed model parameters

| **Parameter** | **Value** | **Units (where applicable)** |
| --- | --- | --- |
| Number of neurons | 100 |  |
| Input firing rate | 100 | Hz |
| External input scaling factor | 21 |  |
