# Supplementary figures and images for: Continual familiarity decoding from recurrent connections in spiking networks
Source: PLoS Comput Biol. 2025 Aug 1;21(8):e1013304. doi: 10.1371/journal.pcbi.1013304 (PMC12334059; doi:10.1371/journal.pcbi.1013304)

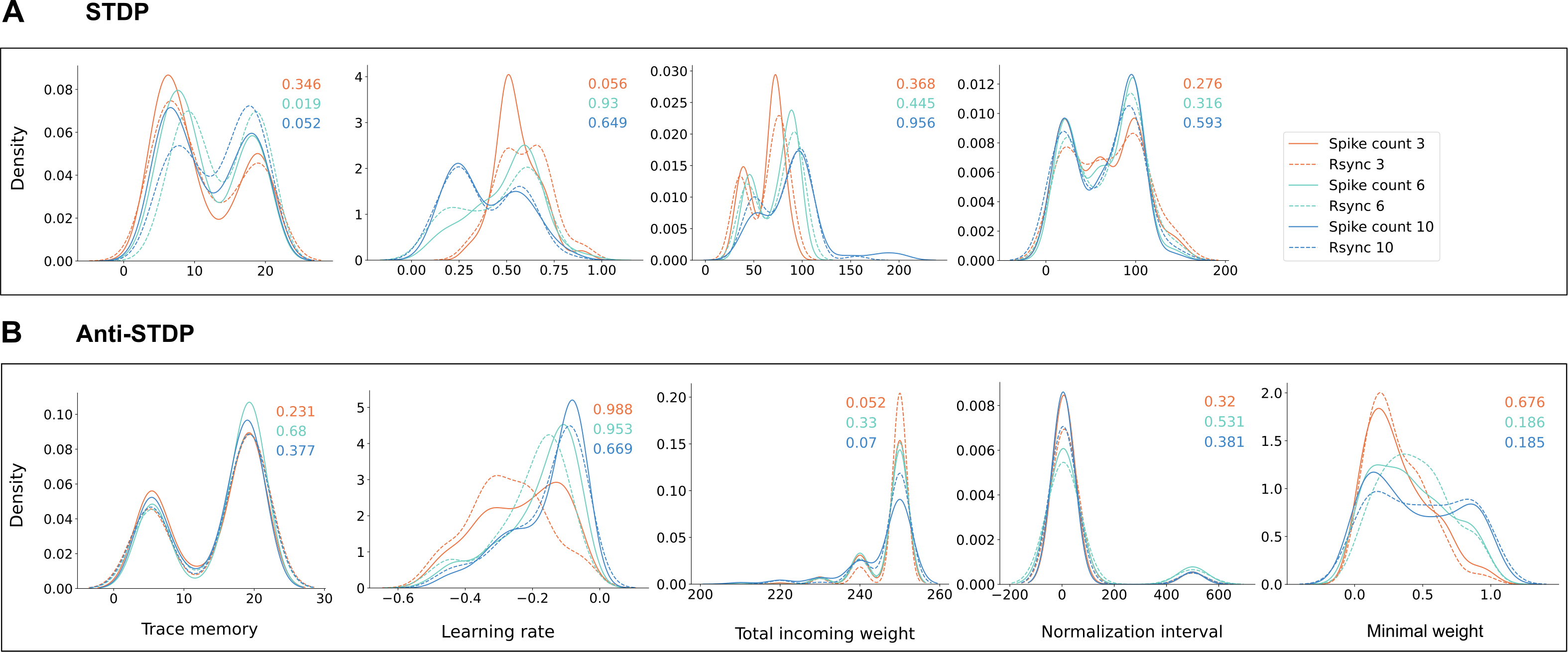

Supplement: S1 Fig — Parameter distributions more strongly differ across repeat intervals than familiarity detection measures ( Rsync and spike count). Each distribution, represented by a colored curve, includes 20 parameter values received during 20 independent optimization procedures. Color stands for repeat interval used during optimization: orange 3, green 6, blue 10. Colored numbers stand for p-values for differences between measures within every sparseness level, computed via a permutation test with 10000 permutations and Bonferroni correction for multiple comparisons. Data for plasticity types: A. Hebbian. B. Anti-Hebbian. No differences between Rsync and spike count-optimal parameters are significant ( p<0.05). (TIF) [file pcbi.1013304.s002.tif]

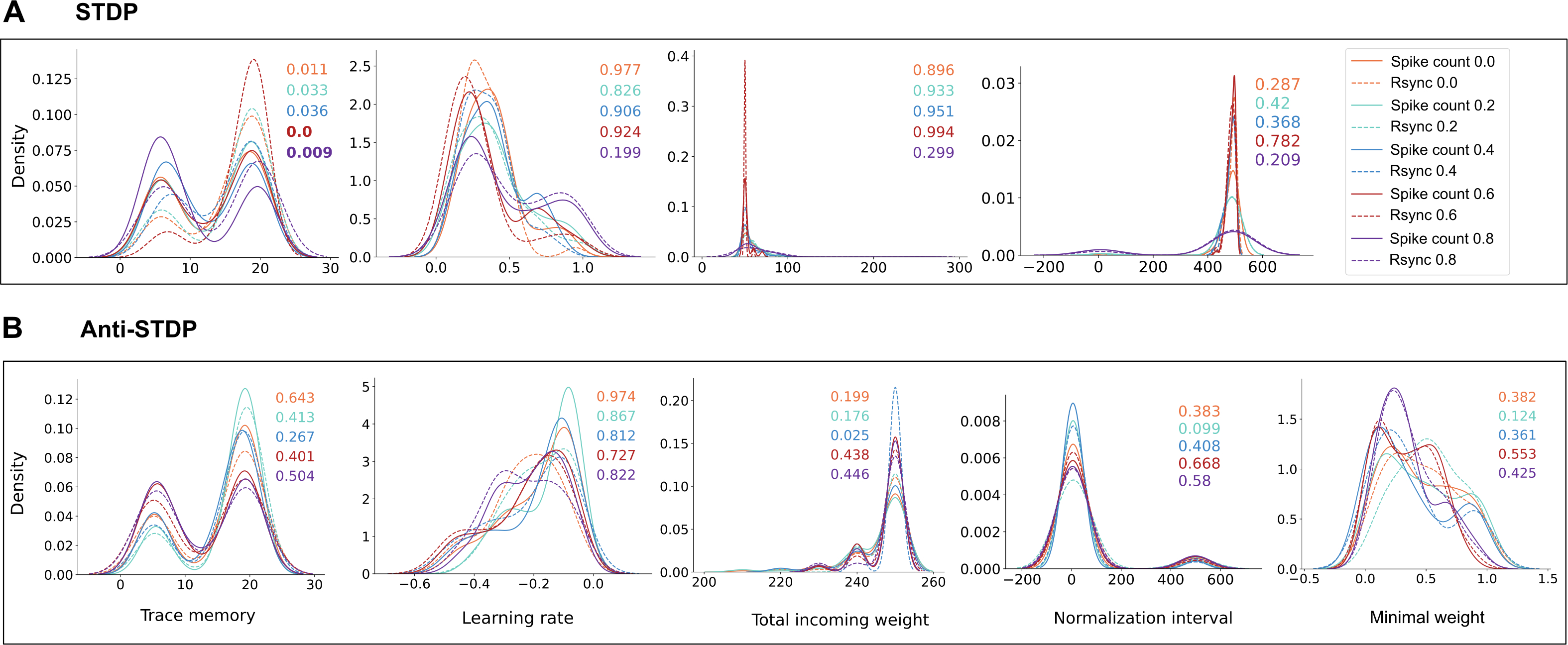

Supplement: S2 Fig — Parameter distributions differ slightly across input template similarity values. Each distribution, represented by a colored curve, includes 10 parameter values received during 10 independent optimization procedures. Color stands for input correlation values: orange 0.0, green 0.2, blue 0.4, red 0.6, purple 0.8. Colored numbers stand for p-values for differences between measures within every sparseness level, computed via a permutation test with 10000 permutations and Bonferroni correction for multiple comparisons. Data for plasticity types: A. Hebbian: values of trace memory are significantly larger for more correlated inputs ( p<0.05). B. Anti-Hebbian. No differences between Rsync and spike count-optimal parameters. (TIF) [file pcbi.1013304.s003.tif]
