# Supplementary material for: Continual familiarity decoding from recurrent connections in spiking networks
Source: PLoS Comput Biol. 2025 Aug 1;21(8):e1013304. doi: 10.1371/journal.pcbi.1013304 (PMC12334059; doi:10.1371/journal.pcbi.1013304)
Supplement: S2 Table — Values of neuron dynamics parameters fixed in all experiments. (DOCX) [file pcbi.1013304.s005.docx]

## S2 Table. Fixed Izhikevich parameters

| **Parameter** | **Value** | **Units (where applicable)** |
| --- | --- | --- |
| Simulation time step | 0.5 | ms |
| Initial membrane potential | 30 | mV |
| Initial recovery variable | 30 | mV |
| Izhikevich a (time scale of recovery variable) | 0.02 |  |
| Izhikevich b (sensitivity of recovery variable) | -0.2 |  |
| Izhikevich c (voltage afterspike reset) | -65.0 | mV |
| Izhikevich d (recovery afterspike update) | 2.0 | mV |
| Excitatory reverse potential | 0.0 | mV |
| Voltage noise amount | 0.6 |  |
| Spike detection threshold | 30 | mV |
| Trace increase | 1.0 |  |
