# Supplementary material for: Continual familiarity decoding from recurrent connections in spiking networks
Source: PLoS Comput Biol. 2025 Aug 1;21(8):e1013304. doi: 10.1371/journal.pcbi.1013304 (PMC12334059; doi:10.1371/journal.pcbi.1013304)
Supplement: Attachment — Submitted filename: PCOMPBIOL-D-25-00093.docx [file pcbi.1013304.s006.docx]

## Overview

This paper presents a novel approach to familiarity detection using a recurrent spiking neural network (SNN) with spike-timing-dependent plasticity (STDP). The study effectively demonstrates that familiarity can be decoded from spike frequency (count) and spike synchrony, with temporal coding showing enhanced performance under sparse input conditions. Furthermore, the proposed model outperforms LSTM in terms of continual familiarity detection and generalization over different time intervals.

The paper is well-written, and provides strong experimental support and in-depth analysis for the claims. However, some areas require clarification and further validation, particularly on the investigation of sparsity, comparison to experimental data and the interpretation of the comparative results to LSTM.

## Strengths

1. The paper is very clear. I appreciate that the authors decided to move much of the mathematical contents to the appendix and keep only the most important ones in the main text.
2. The analysis of the STDP parameters is very comprehensive and provides good understanding of the inner mechanisms of the model.
3. The ability of the SNN to generalize across different repeat intervals is particularly interesting and highlights its potential advantages over traditional machine learning approaches.

## Weaknesses/questions

1. **Correlated patterns**

- **The paper discussed the impact of sparseness on model performance. However, another related, though different property of the patterns, namely pattern correlations, is missing.**
- **Pattern correlation has been shown to be a very important factor influencing familiarity detection (see Bogacz and Brown 2003 for the influence and a way of generating correlated binary patterns). I wonder if the authors could perform a set of experiments on the impact of pattern correlation on the SNN’s performance, which could connect it more closely to previous studies.**

2. **Dependence on Sparsity & Repetition Enhancement**

- The paper claims that **repetition enhancement (increased firing for familiar stimuli)** aligns with **V1 activity**, while **repetition suppression (decreased firing)** is characteristic of regions such as the **perirhinal cortex**.
- However, the authors themselves acknowledge that **V1 typically receives dense inputs**, whereas **the SNN performs worse with dense inputs**. This **contradiction** raises questions about whether the model truly aligns with **V1 physiology**.
- **Suggested Improvement**:
  - A **more thorough review of V1 input sparsity** would strengthen the argument that the SNN could serve as a good model for V1.
  - Alternatively, the authors could **implement anti-Hebbian learning** to test whether the SNN can **exhibit repetition suppression** without compromising performance. If successful, this would make the model a **stronger candidate for modeling the perirhinal cortex**.

3. **Lack of Comparison to Experimental Data**

- The paper mainly focuses on **performance evaluation and connectivity analysis** but lacks a **comparison to biological data**. For example, can this model be **directly compared** to neural recordings of **repetition enhancement in V1**?
- **Suggested Improvement**:
  - The authors could **qualitatively or quantitatively compare** the model’s output to **existing V1 datasets**.
  - Even if an exact match is unavailable, referencing **empirical studies** with **similar spiking patterns** would strengthen the paper’s **biological validity**.
- Including these investigations could improve the biological relevance of the paper and make it a more suitable publication at PLOS computational biology.

4. **Interpreting the Advantage Over LSTM**

- The paper highlights that **LESH generalizes better across repeat intervals than LSTM**. However, is this simply because **LESH and HebbFF continuously update their weights**, while **LSTM’s weights are fixed after training**?
- **Suggested Improvement**:
  - The authors should clarify whether **LESH’s generalization ability is an intrinsic property of STDP or just a consequence of continuous weight updating**.
  - A possible **control experiment** could involve **freezing the LESH weights** after initial learning and testing if it still outperforms LSTM.

5. Explanation of Rsync & Its Role in the Paper

- The paper presents two familiarity decoding strategies: spike count and Rsync (spike synchrony). However, it is unclear why these two methods are compared, as they are external to the SNN itself.
- Key Question: Could these decoding methods equally apply to other familiarity models, and if so, does their comparison belong in this paper?
- Suggested Improvement:
  - A more intuitive explanation of Rsync would help readers understand its significance, instead of a direct explanation of equation 11.
  - The authors should clarify whether the goal is to compare familiarity decoding strategies or evaluate the SNN itself.
  - If Rsync is not specific to SNNs, it might be better suited as a supplementary analysis rather than a main comparison.

References

Bogacz, Rafal, and Malcolm W. Brown. "An anti-Hebbian model of familiarity discrimination in the perirhinal cortex." *Neurocomputing* 52 (2003): 1-6.
