# Supplementary material for: Continual familiarity decoding from recurrent connections in spiking networks
Source: PLoS Comput Biol. 2025 Aug 1;21(8):e1013304. doi: 10.1371/journal.pcbi.1013304 (PMC12334059; doi:10.1371/journal.pcbi.1013304)
Supplement: Attachment — Submitted filename: ReviewPlos.docx [file pcbi.1013304.s007.docx]

- What are the main claims of the paper and how significant are they for the discipline?

The paper shows how a recurrent spiking network can simulate familiarity, defined here as the capacity to recognize a previously encountered stimulus. It also studies variables that modulate the model efficiency and compare the presented model to 2 others computational models.

- Are these claims novel? If not, which published articles weaken the claims of originality of this one?

What is novel is the use of spiking neurons.

- Are the claims properly placed in the context of the previous literature? Have the authors treated the literature fairly?

The review should be extended to include studies on cognition, computational models, electrophysiological findings, and imagery. Additionally, it should provide a more comprehensive exposition of the psychological phenomenon of familiarity, including its relationship to memory and the distinctions between relative and absolute familiarity.

- Do the data and analyses fully support the claims? If not, what other evidence is required?

OK

- Would additional work improve the paper? How much better would the paper be if this work were performed and how difficult would it be to do this work?

Yes

The authors used an oversimplified STDP rule, resembling a spike timing correlation rule.

In a Spike Timing Dependent Plasticity process, if the presynaptic neuron fires before the postsynaptic spike, the connection is potentiated. Conversely, if the presynaptic neuron fires after or at the same time as the postsynaptic spike, the connection is depressed.

It would be beneficial to use a rule that better matches STDP, incorporating both its Hebbian and anti-Hebbian components.

Several papers claim that anti-Hebbian learning more effectively models familiarity. In our paper, we even hypothesized that Hebbian and anti-Hebbian rules could be complementary in addressing different aspects of familiarity.

Adding an anti-Hebbian component to your spiking neuron model (LESH) would offer a better comparison to HebbFF, which combines Hebbian and anti-Hebbian learning with a feedforward network.

This addition would also eliminate the need for the blind normalization procedure described in equation 3, which you use to prevent uncontrollable increases in connection weights.

- *PLOS Computational Biology* encourages authors to publish detailed protocols and algorithms as supporting information online. Do any particular methods used in the manuscript warrant such treatment? If a protocol is already provided, for example for a randomized controlled trial, are there any important deviations from it? If so, have the authors explained adequately why the deviations occurred?

OK github doi

- Are original data deposited in appropriate repositories and accession/version numbers provided for genes, proteins, mutants, diseases, etc.?

Yes github doi

- Has the author-generated code that underpins the findings been made publicly available?

Yes

- Does the study conform to any [relevant guidelines](https://journals.plos.org/ploscompbiol/s/best-practices-in-research-reporting#loc-reporting-guidelines-for-specific-research-resources-and-study-types) such as CONSORT, MIAME, QUORUM, STROBE, and the [Fort Lauderdale agreement](https://www.sanger.ac.uk/wp-content/uploads/fortlauderdalereport.pdf)?

Don’t know

- Are details of the methodology sufficient to allow the experiments to be reproduced?

Almost I would change some equations…

For example in eq 1 and 2, if I do understand well, the letter i stand for a particular neuron in Ai or wij and a particular time in ti. It would be better to use different letters. Or if I do not understand you need to improve the explanation.

In the equation 11, I do not see why you use activation A as a measure of synchrony, instead of neuron spikes. Since you need to measure spike synchrony. This should be explained.

- Is any software created by the authors freely available?

Python programs yes

- Is the manuscript well organized and written clearly enough to be accessible to non-specialists?

It needs to be improved in the introduction. I suggest improving literature review and better emphasize the psychological phenomena of familiarity vs recollection…

- Does the paper use standardized scientific nomenclature and abbreviations? If not, are these explained at the first usage?

OK
