# Supplementary material for: Continual familiarity decoding from recurrent connections in spiking networks
Source: PLoS Comput Biol. 2025 Aug 1;21(8):e1013304. doi: 10.1371/journal.pcbi.1013304 (PMC12334059; doi:10.1371/journal.pcbi.1013304)
Supplement: Attachment — Submitted filename: Response to Reviewers.docx [file pcbi.1013304.s009.docx]

# **Response to Editors**

Dear Prof. Dr. Bogacz and Prof. Dr. Ayers,

We greatly appreciate your interest in our manuscript and your thoughtful advice on its improvement.

Following your suggestion and suggestions of the reviewers, we included a separate analysis for correlated patterns in our study: please see Fig. 4, L373-393 for result and L863-884 for methodological details.

We also conducted one additional analysis suggested by reviewers: performance of an anti-Hebban recurrent network on a continual familiarity task. We repeated all experiments with an anti-Hebbian learning algorithm and updated Fig. 1-6 and their interpretation in text (L335-336, 357-366, 433-504, 561-579) in the Results section. The implementation details can be found in Fig. 1, Eq. 3 and L136-142.

In addition to new experiments, we extended our Introduction (L54-58, 64-92) and Discussion (L646-665, 699-723) sections, which provides better theoretical context from the psychological and physiological point of view following the reviewers’ suggestions. Namely, we extended our literature review to include studies on dual-process theory, relationship between recollection and familiarity, relative and absolute familiarity classification, neural correlates of familiarity, and interpretation of our results in the context of experimental findings.

During the revision process, we also identified inconsistencies in the description of our methodology and updated those. Please see the corrected description of a unidirectional STDP algorithm in Eq. 2 and L129-135, and the corrected list of fixed optimization parameters in Supplementary Table S4.

The rest of this letter is dedicated to detailed individual responses to the reviewers' comments.

# **Response to Reviewer 1**

Dear Reviewer 1,

Thank you for your thorough and insightful review of our manuscript. We are glad to hear that you found our writing clear, since we indeed put a lot of thought into the organization of mathematical sections in the text. Also, we greatly appreciate your recognition of the interesting nature of our STDP parameter analysis and generalizability over repeat intervals.

## 1. Correlated patterns

We found your idea to conduct an experiment on pattern correlations very interesting, considering that it falls in line very well with our exploration of repeat intervals and sparseness levels. Following the proposed reference, we included this analysis in our Results section (Fig. 4 and L373-393), with implementation explained in Methods (L863-884).

## 2. Dependence on Sparsity & Repetition Enhancement

Thank you for the comment. It helped us pinpoint that we formulated our understanding of sparseness rather vaguely in the Discussion. We have now clarified it and added a more thorough review of V1 population sparseness activity, as you suggested (L666-674).

In addition, in response to your comments and comments by the second reviewer, we decided to additionally explore anti-Hebbian plasticity in our model and compare its performance to the one of the original Hebbian implementation. Please refer to Figures 1-6, their respective interpretations (L335-336, 357-366, 433-504, 561-579) and implementation details in Fig. 1, Eq. 3 and L136-142. The implementation of the anti-Hebbian model is slightly different than Hebbian, since we needed to introduce additional 2 mechanisms for stabilizing this kind of plasticity in a recurrent network (described in L136-142).

We also extended our analysis of the experimental data on repetition suppression and enhancement in L68-70, 707-720.

## 3. Lack of Comparison to Experimental Data

We appreciate your interest in our model’s comparison to biological data. Although we were not able to find a well-matching dataset, we attempted to do a better qualitative comparison with V1 data in L716-723, as well as provided a more comprehensive analysis of the said V1 study in general. We believe an exact quantitative comparison would go beyond the scope of this paper, but we are looking out for appropriate datasets.

In addition, we extended the analysis of other experimental studies in relation to our model’s results in Discussion (L707-720), as well as included several more experimental studies on familiarity neural correlates in the literature review (L64-80).

Added citations (in the order of mention in References):

Brandt KR, Eysenck MW, Nielsen MK, von Oertzen TJ. Selective lesion to the entorhinal cortex leads to an impairment in familiarity but not recollection. Brain and cognition. 2016 Apr 1;104:82-92.

Bridger EK, Bader R, Mecklinger A. More ways than one: ERPs reveal multiple familiarity signals in the word frequency mirror effect. Neuropsychologia. 2014 May 1;57:179-90.

Voss JL, Federmeier KD. FN400 potentials are functionally identical to N400 potentials and reflect semantic processing during recognition testing. Psychophysiology. 2011 Apr;48(4):532-46.

Duke D, Martin CB, Bowles B, McRae K, Köhler S. Perirhinal cortex tracks degree of recent as well as cumulative lifetime experience with object concepts. Cortex. 2017 Apr 1;89:61-70.

Yang H, McRae K, Köhler S. Perirhinal cortex automatically tracks multiple types of familiarity regardless of task-relevance. Neuropsychologia. 2023 Aug 13;187:108600.

Hayden DJ, Montgomery DP, Cooke SF, Bear MF. Visual recognition is heralded by shifts in local field potential oscillations and inhibitory networks in primary visual cortex. Journal of Neuroscience. 2021 Jul 21;41(29):6257-72.

Ho JW, Poeta DL, Jacobson TK, Zolnik TA, Neske GT, Connors BW, Burwell RD. Bidirectional modulation of recognition memory. Journal of Neuroscience. 2015 Sep 30;35(39):13323-35.

## 4. Interpreting the Advantage Over LSTM

We appreciate your feedback on the lack of the analysis of LSTM performance, this is an important point. We attempted to clarify our interpretation of its poor generalizability in L279-282, 287-296.

Our explanation also provides an insight into why the suggested control experiment would not be informative. LESH and LSTM use fundamentally different strategies to encode memory: LSTM stores past memory representations in hidden states, while LESH stores them directly in recurrent connections with the use of plasticity. Therefore, freezing LESH weights, i.e. turning off the plasticity, would not allow memories to form and essentially rid the model of the very capability to memorize. We address this directly in the text in L279-282.

## 5. Explanation of Rsync & Its Role in the Paper

Following your suggestion, we now provide an intuitive interpretation of the Rsync metric, in addition to the mathematical explanation, in L260-265.

We have also worked on our introduction and the framing of Rsync in the context of our paper to better explain why we included the analysis (L93-97): In recurrent / laterally connected networks, we previously found that Rsync / temporal synchrony can also be used to decode stimulus familiarity with fixed network structures, we wanted to investigate whether this is true for continuous familiarity encoding as well. We believe this brings a new perspective of how familiarity may be encoded in different cortical areas. We highlighted this potential avenue for future research in the Discussion session (L721-723).

# **Response to Reviewer 2**

Dear Prof. Dr. Sougné (Reviewer 2),

We appreciate your thoughtful review, which helped us improve the clarity and thoroughness of our manuscript. We are glad to know that you found our experiments and analysis sound and novel.

## 1. Extend Review

Following your suggestion, we have enriched the analysis of relevant literature in Introduction and Discussion sections. We added information on relative and absolute familiarity (L64-80), expanded the review of related simulation studies (L81-89) and provided a more comprehensive comparison to existing familiarity models (L711-713).

We additionally cited 9 relevant studies (in the order of mention in References):

Wolk DA, Dunfee KL, Dickerson BC, Aizenstein HJ, DeKosky ST. A medial temporal lobe division of labor: insights from memory in aging and early Alzheimer disease. Hippocampus. 2011 May;21(5):461-6.

Brandt KR, Eysenck MW, Nielsen MK, von Oertzen TJ. Selective lesion to the entorhinal cortex leads to an impairment in familiarity but not recollection. Brain and cognition. 2016 Apr 1;104:82-92.

Bridger EK, Bader R, Mecklinger A. More ways than one: ERPs reveal multiple familiarity signals in the word frequency mirror effect. Neuropsychologia. 2014 May 1;57:179-90.

Voss JL, Federmeier KD. FN400 potentials are functionally identical to N400 potentials and reflect semantic processing during recognition testing. Psychophysiology. 2011 Apr;48(4):532-46.

Duke D, Martin CB, Bowles B, McRae K, Köhler S. Perirhinal cortex tracks degree of recent as well as cumulative lifetime experience with object concepts. Cortex. 2017 Apr 1;89:61-70.

Yang H, McRae K, Köhler S. Perirhinal cortex automatically tracks multiple types of familiarity regardless of task-relevance. Neuropsychologia. 2023 Aug 13;187:108600.

Hayden DJ, Montgomery DP, Cooke SF, Bear MF. Visual recognition is heralded by shifts in local field potential oscillations and inhibitory networks in primary visual cortex. Journal of Neuroscience. 2021 Jul 21;41(29):6257-72.

Ho JW, Poeta DL, Jacobson TK, Zolnik TA, Neske GT, Connors BW, Burwell RD. Bidirectional modulation of recognition memory. Journal of Neuroscience. 2015 Sep 30;35(39):13323-35.

Kazanovich Y, Borisyuk R. A computational model of familiarity detection for natural pictures, abstract images, and random patterns: Combination of deep learning and anti-Hebbian training. Neural Networks. 2021 Nov 1;143:628-37.

Read J, Delhaye E, Sougné J. Computational models can distinguish the contribution from different mechanisms to familiarity recognition. Hippocampus. 2024 Jan;34(1):36-50.

Bogacz R, Brown MW. An anti-Hebbian model of familiarity discrimination in the perirhinal cortex. Neurocomputing. 2003 Jun 1;52:1-6.

## 2. Asymmetrical (classical) STDP

We thank you for this thoughtful comment.

First of all, it helped us identify the existing inconsistencies in method descriptions. We indeed used a unidirectional Hebbian model for our initial simulations. Our motivation for using this simplified plasticity rule was based on the nature of our input stimuli, which are purely rate-encoded and lack temporal structure. Canonical bidirectional STDP rules with LTP+LTD components are well suited for learning temporally structured patterns, therefore with our input structure we decided to follow the approach of prior work (e.g., 24, 26, 34 in References) and focused on a simplified Hebbian update. This decision allowed us to isolate the effects of activity-dependent strengthening in a clean and interpretable manner.

However, we fully agree that anti-Hebbian plasticity is a well-established direction for familiarity modeling, especially after exploring more literature on repetition suppression in relative familiarity, as you recommended in your previous comment. Therefore, we decided to explore unidirectional negative-only recurrent anti-Hebbian plasticity in our model.

In order to be able to conduct a straightforward comparison with our positive-only Hebbian model, we implemented a negative-only rule, as in Ref. 23, 24, 25, 26. Results are presented in comparison with the Hebbian model in Fig. 1-6 and in the corresponding text (L335-336, 357-366, 433-504, 561-579). The implementation details are described in L136-142. Please note that the implementation of the anti-Hebbian model is slightly different than Hebbian, since we needed to introduce additional 2 mechanisms for stabilizing this kind of plasticity in a recurrent network (also described in L136-142).

We are grateful for encouraging this extension, which we believe substantially broadened our study with interesting computational comparisons and implications for feedforward and recurrent familiarity coding.

## 3. Equations

Following your question, we clarified Equations 1-2 (and added Eq. 3). We also changed the notation in Eq. 6 and new Eq. 5.

We appreciate your interest in our use of smoothed activation traces for computing synchrony. Rather than measuring strict spike-time coincidences, Rsync metric is designed to capture *functional synchrony*, i.e. the degree to which neurons are co-active within a short temporal window. To this end, we convolve the binary spike trains with an exponential kernel to generate activation signal A, which reflects the temporally smoothed influence of each spike. This approach provides two advantages: 1) it increases robustness to small timing differences (jitter) between neuron spikes, and 2) it yields a continuous measure of coactivation that is more biologically plausible, as real neurons integrate input over time rather than responding to isolated, instantaneous spikes.

This technique is useful when analyzing synchrony in the absence of perfectly aligned spike timing, as in rate-coded or noisy spiking regimes, such as our inherently noisy Poisson input encoding.

## 4. Introduction

As per comment 1, we substantially increased our review of relevant works. We made sure to introduce the phenomenon of recollection and its relation to familiarity in L52-58.

We additionally cited 2 relevant studies (in the order of mention in References):

Diana RA, Reder LM, Arndt J, Park H. Models of recognition: A review of arguments in favor of a dual-process account. Psychonomic bulletin & review. 2006 Feb;13(1):1-21.

Scalici F, Caltagirone C, Carlesimo GA. The contribution of different prefrontal cortex regions to recollection and familiarity: a review of fMRI data. Neuroscience & Biobehavioral Reviews. 2017 Dec 1;83:240-51.
