# Supplementary material for: Continual familiarity decoding from recurrent connections in spiking networks
Source: PLoS Comput Biol. 2025 Aug 1;21(8):e1013304. doi: 10.1371/journal.pcbi.1013304 (PMC12334059; doi:10.1371/journal.pcbi.1013304)
Supplement: Attachment — Submitted filename: Response_to_Reviewers_auresp_2.docx [file pcbi.1013304.s010.docx]

# **Response to Editors**

Dear Prof. Dr. Bogacz and Prof. Dr. Ayers,

Thank you for your kind acknowledgement of the revisions. We are also grateful for your careful reading, which helped us catch subtle issues that we previously overlooked.

Following your suggestions, we corrected references in L89-90 and L865-866, as well as updated reference 27 (L1041). We also unified HebbFF model mentions at L268-274 and corrected the mathematical symbol at L126.

To improve clarity of the anti-STDP results, we explicitly mentioned that the results were produced by the anti-Hebbian version of our LESH implementation in L357, L389, L586. Another clarity improvement included simplifying the correlation cartoon in Fig. 1C, as per your suggestion. We indeed did not explore -1 correlation in our study.

Finally, we corrected an inconsistency in sparseness description in L189-191. Now it properly introduces our definition of sparseness, which is further detailed in L749-758.

# **Response to Reviewer 2**

Dear Reviewer 2,

Thank you for recognizing the effort we put into addressing the reviewers’ suggestions. We greatly appreciate your contribution to strengthening the manuscript.

As per your careful observations, we corrected the character display at L126, fixed the typos in L527 and L753, as well as improved the coloring scheme in Fig. 2C.

After re-reading the manuscript, we agree that the paragraph on page 27 may sound confusing. While we have chosen to keep Figures S2 and S3 in the Supplementary Materials to avoid distracting from the main message of the paper, we have revised the paragraph to improve clarity: please see L580-589.
